# Association between circulating 25-hydroxyvitamin D and cardiometabolic risk factors in adults in rural and urban settings

**DOI:** 10.1038/s41387-022-00215-1

**Published:** 2022-07-29

**Authors:** Camille M. Mba, Albert Koulman, Nita G. Forouhi, Stephen J. Sharp, Fumiaki Imamura, Kerry Jones, Sarah R. Meadows, Felix Assah, Jean Claude Mbanya, Nick J. Wareham

**Affiliations:** 1 MRC Epidemiology Unit, University of Cambridge School of Clinical Medicine, Cambridge, United Kingdom; 2 Department of Public Health, Faculty of Medicine and Biomedical Sciences, University of Yaoundé 1, Yaoundé, Cameroon; 3 National Institute for Health Research Biomedical Research Centre Nutritional Biomarker Laboratory, University of Cambridge School of Clinical Medicine, Cambridge, United Kingdom; 4 Department of Internal Medicine and Specialties, Faculty of Medicine and Biomedical Sciences, University of Yaoundé 1, Yaoundé, Cameroon

**Keywords:** Endocrine system and metabolic diseases, Hormones

## Abstract

**Background:**

An inverse association between vitamin D status and cardiometabolic risk has been reported but this relationship may have been affected by residual confounding from adiposity and physical activity due to imprecise measures of these variables. We aimed to investigate the relationship between serum 25-hydroxyvitamin D (25(OH)D) and cardiometabolic risk factors, with adjustment for objectively-measured physical activity and adiposity.

**Methods:**

This was a population-based cross-sectional study in 586 adults in Cameroon (63.5% women). We assessed markers of glucose homoeostasis (fasting blood glucose (BG), 2 h post glucose load BG, HOMA-IR)) and computed a metabolic syndrome score by summing the sex‐specific z‐scores of five risk components measuring central adiposity, blood pressure, glucose, HDL cholesterol and triglycerides.

**Results:**

Mean±SD age was 38.3 ± 8.6 years, and serum 25(OH)D was 51.7 ± 12.5 nmol/L. Mean 25(OH)D was higher in rural (53.4 ± 12.8 nmol/L) than urban residents (50.2 ± 12.1 nmol/L), *p* = 0.002. The prevalence of vitamin D insufficiency (<50 nmol/L) was 45.7%. There was an inverse association between 25(OH)D and the metabolic syndrome score in unadjusted analyses (β = −0.30, 95% CI −0.55 to −0.05), which became non-significant after adjusting for age, sex, smoking status, alcohol intake and education level. Serum 25(OH)D was inversely associated with fasting BG (−0.21, −0.34 to −0.08)), which remained significant after adjustment for age, sex, education, smoking, alcohol intake, the season of data collection, BMI and physical activity (−0.17, −0.29 to −0.06). There was an inverse association of 25(OH)D with 2-h BG (−0.20, −0.34 to −0.05) and HOMA-IR (−0.12, −0.19 to −0.04) in unadjusted analysis, but these associations became non-significant after adjustment for potential confounders.

**Conclusion:**

Vitamin D insufficiency was common in this population. This study showed an inverse association between vitamin D status and fasting glucose that was independent of potential confounders, including objectively measured physical activity and adiposity, suggesting a possible mechanism through insulin secretion.

## Introduction

The burden of cardiometabolic diseases has risen over the last decades in sub-Saharan Africa (SSA) where the highest age-standardized mortality rates occur and therefore is of growing public health concern [1]. Vitamin D is suggested to play a role in the prevention of cardiometabolic diseases, but evidence from observational studies of the relationship between vitamin D status and cardiometabolic diseases is inconsistent [2–4]. A meta-analysis of 38 cross-sectional studies and 5 longitudinal studies showed an inverse association between serum 25-hydroxyvitamin D (25(OH)D) concentration and metabolic syndrome in cross-sectional studies but not in longitudinal studies [5]. However, there was evidence of high between-study heterogeneity (*I*^2^ > 90%) and few studies included in the review were population-based. Therefore, the association between vitamin D status and metabolic syndrome remains unclear.

Accumulating evidence from observational studies suggests the association between vitamin D and cardiometabolic outcomes may depend on the prevalence of low vitamin D status in the population studied [6, 7]. Although most randomized controlled trials (RCTs) suggest no benefit of vitamin D supplementation on cardiometabolic outcomes, these trials were conducted in participants that are in majority replete with vitamin D [8, 9]. Thus, the context in which an association between vitamin D and cardiometabolic disease is observed may be critical and outstanding questions remain about the role of vitamin D on cardiometabolic outcomes in populations that are inadequate for vitamin D.

Data suggest that vitamin D insufficiency is a public health issue in many SSA countries. A meta-analysis of data from 23 African countries found low concentrations of serum 25(OH)D ( < 30 nmol/L) in 18.5% of the population [10]. This compares with a global prevalence of 6.7% (25(OH)D < 25 nmol/L) [11] and a prevalence of 13.0% (25(OH)D < 30 nmol/L) in Europe [12]. Despite the high burden of vitamin D deficiency in SSA, we found only two studies from South Africa in urban populations only that investigated the link between vitamin D status and metabolic syndrome and reported no evidence of an association with the metabolic syndrome or individual metabolic risk factors [13, 14].

The inverse association between vitamin D status and cardiometabolic risk factors reported in previous studies might be the result of residual confounding due to imprecise measurements of confounding factors. Amongst the potential confounders in the association of vitamin D status and cardiometabolic risk factors, physical activity and adiposity are some of the most challenging to measure and are highly susceptible to errors. The clarification of the association between vitamin D status and metabolic syndrome requires objective measures of physical activity and more precise measures of adiposity. Most previous observational studies either did not account for physical activity or recorded only self-reported physical activity, which is subject to measurement error and recall bias, thus creating the potential for residual confounding even after adjustment. Physical activity has been reported to be correlated with serum 25(OH)D concentrations, with stronger associations observed for objectively-measured physical activity than self-report.

We conducted this study to address the research gap in the literature on the association between vitamin D status and cardiometabolic risk factors with adjustment for objectively-measured physical activity and adiposity in SSA, a region with a high burden of vitamin D insufficiency and cardiometabolic disease [10, 15, 16].

## Methods

### Study design and participants

The methods used for data collection for this cross-sectional population-based study have been described in detail elsewhere [17]. Briefly, adults aged 25 to 55 years without a history of diabetes or cardiovascular disease were recruited in four sites in Cameroon: two rural (Bafut and Mbankomo) and two urban residential areas (Bamenda and Yaoundé) in the North-west region and Centre regions of Cameroon. All adults aged 25 to 55 years without a history of diabetes or cardiovascular disease were approached through door-to-door recruitment. A total of 651 participants (rural: *n* = 303, mean age 38.5 ± 8.3 years; urban: *n* = 348, mean age 37.9 ± 9.1 years) agreed to take part in this study. The mean age and sex ratio of volunteers was similar to all 3854 eligible participants identified in the delimited areas.

Ethical approval for this study was obtained from the Cameroon National Ethics Committee, and all participants provided written informed consent before inclusion. A total of 586 participants had serum samples available for 25(OH)D measurements.

### Socio-demographic and anthropometric measurements

Data were collected over a period of 15 months. At inclusion, self-reported information was collected on socio-demographic (age, sex, level of education, residential site) and behavioural characteristics (alcohol intake, smoking, physical activity, fruit and vegetable intake) using an adapted version of the World Health Organization (WHO) STEPwise Approach to Surveillance questionnaire. Smoking status and alcohol drinking were categorised as never, past or current, based on responses to the questions “have you ever smoked any tobacco product/consumed a drink that contains alcohol?” and “do you currently smoke any tobacco product/did you consume a drink that contains alcohol within the past 12 months?”. Waist circumference (WC) was measured to the nearest 0.1 cm using a non-stretch fibreglass tape at the level of the midpoint between the lower costal margin and the anterior superior iliac crests of participants wearing light clothing. Central obesity was defined as waist circumference ≥80 cm in women or ≥94 cm in men. Bodyweight and composition were measured using electronic scales and bio-impedance (Tanita TBF-531 scales; Tanita UK, Uxbridge, Middlesex, U.K.), respectively. Height was measured in individuals without shoes and belts using a standard rigid stadiometer and body mass index (BMI in kg/m^2^) computed as the body weight (kg) divided by the square of height (m^2^). Where relevant the following cut-offs were used to categorise BMI: less than 18.5, underweight; 18.5−24.9, normal weight; 25−29.9, overweight and ≥30 kg/m^2^, obese.

Blood pressure was measured on the dominant arm of the participants after at least 5 minutes of rest using automated blood pressure (BP) measuring device (OMRON M4-I). Three measurements of BP were taken at 1-minute intervals and the BP value was computed as the average of the three recordings.

Both self-reported and objectively measured physical activity data were collected from all the participants. Using the global physical activity questionnaire (GPAQ), we recorded self-reported activities at work, recreational activities and travel and derived estimates of energy expenditure in each domain in MET-min/week and physical activity energy expenditure (GPAQ PAEE). Objectively measured physical activity energy expenditure (PAEE) was measured using a combined heart rate (HR) and movement sensor (Actiheart; Cambridge Neurotechnology, Cambridge, U.K.) over seven continuous days. We used individual HR responses during a step test for the individual calibration of HR data. This method, previously validated against the criterion of doubly labelled water in this population (*r* = 0.40), is detailed elsewhere [18]. PAEE was scaled for body weight and expressed as KJ/Kg/day. Categories were created based on time spent in minutes per day at different intensities of physical activity: <1.5 METS, sedentary behaviour; 1.5–3 METS, light physical activity (LPA); > 3 METS, moderate to vigorous physical activity (MVPA). Throughout, we use PAEE to refer to objectively measured physical activity.

### Biochemical measurements

All participants provided blood samples in the morning between 7:30 and 9:30 AM after an overnight fast of at least 8 hours. Following centrifugation at ~1400 *g*, plasma and serum were aliquoted and stored at −80 °C. Frozen samples were transported by air on dry ice to Cambridge, UK and stored at −80 °C until analysis. Participants underwent a 75 g oral glucose tolerance test; with glucose measured on fresh capillary whole blood using a Hemocue B-Glucose Analyzer (HemoCue AB, Ängelholm, Sweden) before (fasting) and 2 h post glucose load.

Fasting plasma insulin was assayed in singleton by two-step fluorometric assay on a 1235 AutoDELFIA automatic immunoassay system (kit by Perkin Elmer Life Sciences; Wallac Oy, Turku, Finland). Triglycerides, HDL and total cholesterol were measured by the enzymatic method according to the manufacturer’s instructions using automated assays on the Dade Behring Dimension RxL analyzer. LDL cholesterol concentrations were derived by the Friedewald formula (LDL cholesterol = total cholesterol − (triglyceride/2.2) – HDL), when triglyceride concentration was <4.5 mmol/L. Measurement of C-reactive protein (CRP) was automated based on the particle-enhanced turbidimetric-immunoassay (PETIA) technology using the Dade Behring Dimension RxL analyzer. Samples were analyzed at the National Institute for Health Research (NIHR) Cambridge Biomedical Research Centre (BRC), Core Biochemical Assay Laboratory.

### Measurement of serum 25(OH)D

Serum 25(OH)D concentrations were measured at the NIHR Cambridge BRC Nutritional Biomarker Laboratory (NBL) using ultra-high-performance liquid chromatography-tandem mass spectrometry (UPLC-MS/MS). Quality assessment of the assay was performed regularly as part of the Vitamin D External Quality Assessment Scheme (www.deqas.org) and performance was additionally assessed against National Institute of Standards and Technology Standard Reference Material 972a.

Serum samples stored at −80 °C were removed and thawed, and stable isotope-labelled internal standards were added to normalize the sample preparation process and instrument detection variability. Protein precipitation was performed with methanol followed by liquid-liquid extraction in hexane. Samples were dried and then reconstituted in 73% methanol. Samples were injected onto the UPLC (Waters Acquity, Waters UK, Herts, UK) and chromatographic separation was performed with a Hypersil GOLD PFP 2.1 x 100 mm 1.9 µm column (Thermo Scientific, UK) at 40 °C and with a 74% methanol +0.1% formic acid isocratic mobile phase at 0.25 mL/min. Detection was accomplished by an AB Sciex QTrap 4000 mass spectrometer (AB Sciex, Warrington, UK). The ratio of the signal of the metabolite to the internal standard obtained was compared against that of a calibration curve to determine the concentration of 25(OH)D_2_, 25(OH)D_3_ and C3 epimer of -25(OH)D_3_ (epi-25(OH)D_3)_.

Total of 25(OH)D was calculated from the sum of 25(OH)D_2_ and 25(OH)D_3_ and was used in subsequent data analysis. The inter-assay coefficient of variation for all metabolites ranged between 5.1% and 7.8%. The limit of detection (LOD) was 2, 3 and 4 nmol/L for 25(OH)D_3_, 25(OH)D_2_ and epi-25(OH)D_3_ respectively. The limit of quantification (LOQ) was 6 nmol/L for all the metabolites. Over 90% of participants had non-detectable levels of 25(OH)D_2_. We imputed a random number between 3 and 6 for 25(OH)D_2_ results above the LOD but below the LOQ (n = 27).

### Outcomes

Outcomes were a continuous metabolic syndrome score and markers of glucose homoeostasis (fasting glucose, 2 h glucose and homoeostatic model assessment of insulin resistance (HOMA-IR)). HOMA-IR was computed using the formula = ([FPI × FBG]/22.5)), where FPI is fasting plasma insulin (mU/L) and FBG is fasting blood glucose (mmol/L) [19]. We derived a continuous metabolic syndrome score based on the five risk factors (waist circumference, fasting blood glucose, blood pressure, triglycerides and HDL cholesterol) in the definitions of the National Cholesterol Education Program Adult Treatment Program III. We used a continuous score instead of a binary definition to maximise statistical power to detect associations. Moreover, there is increasing evidence supporting the use of a continuous metabolic syndrome score in epidemiological research [20, 21]. The metabolic syndrome score was calculated by summing sex-specific standardized continuous values of central obesity (waist circumference), glycemia (fasting blood glucose), mid blood pressure [(systolic blood pressure + diastolic blood pressure)/2] [22] and blood lipids (triglycerides and HDL cholesterol with the latter coded in an opposite direction to the other factors; i.e inverted HDL). We standardized each of the five factors by subtracting the sample mean from individual values and dividing it by the standard deviation of the sample mean. Triglycerides was log-transformed to meet the normality assumption. The five components of the metabolic syndrome were equally weighted in the calculation. A higher score is indicative of a less favourable metabolic syndrome profile. In parallel, we computed the metabolic syndrome score without waist circumference in a sensitivity analysis.

### Statistical analysis

Statistical analyses were performed using Stata 15 (StataCorp, Texas, United States). Descriptive statistics are presented as means and standard deviations (or median and [25−75th percentile] for non-normally distributed data) or numbers and percentages.

Using the date of blood draw, we derived the season of measurement of 25(OH)D as long dry (December-March), light rain (April-May), short dry (June−July), heavy rain (August−November). We reported the proportion of participants with deficient, insufficient and adequate vitamin D status using cut-offs suggested by the Institute of Medicine (deficient <30 nmol/L, inadequate but not deficient: 30−49.9 nmol/L, adequate: 50−74.9 nmol/L) [23] and optimal vitamin D status as suggested by the Endocrine Society (≥75 nmol/L) [24]. We tested differences in means using the *t*-test (or differences in medians using the Mann−Whitney test) and differences in proportions using the chi-squared test. Linear trends across quartiles of serum 25(OH)D concentrations were assessed by linear regression for continuous variables and chi-squared test for trend for categorical variables (Cochran-Armitage test or Cochran-Mantel-Haenszel test for categorical variables with 2 or ≥3 levels respectively).

Multiple linear regression adjusted for age and sex was used to identify predictors of continuously distributed serum 25(OH)D. To estimate associations with outcome variables, we fitted 5 models that were incrementally adjusted to account for potential confounding variables based on biological plausibility and previous research. Model 1 was unadjusted, model 2 was adjusted for age (continuous), sex, smoking (never, past or current), alcohol intake (never, past or current) and level of education (less than primary school, completed primary school, secondary school and university). Model 3 was additionally adjusted for the season of blood draw (long dry, short rainy, short dry and long rainy), residential site (4 sites). Model 4 was additionally adjusted for BMI (continuous) and model 5 for objectively measured PAEE (continuous). We investigated non-linear associations of serum 25(OH)D with outcomes by fitting restricted cubic splines with 5 knots corresponding to the 5th, 27.5th, 50th, 72.5th and 95th percentile of continuous serum 25(OH)D. As there was no evidence of a non-linear association with any of the outcomes, we fitted linear regression models. With missing information observed, complete-case analyses were performed for metabolic syndrome score (*n* = 528) and the other glycemic markers (*n* = 530−537) with further sensitivity analysis implementing multiple imputations.

We tested interactions of 25(OH)D with sex, rural-urban residence and BMI categories (normal weight, overweight and obese) using model 5. When the *p*-value for interaction was <0.05, we reported results within the relevant strata. We performed sensitivity analyses (a) using multiple imputations to investigate the impact of missing data. With less than 10% of missing data, missing data were imputed under the assumption of missing at random with multiple imputations by chained equations to create 10 imputed datasets and combining estimates across the imputed datasets. (b) Replaced BMI by body fat in model 4 and 5; (c) included self-reported fruit and vegetable intake as a proxy for overall dietary quality in model 5 (d) used a metabolic syndrome score calculated without the waist circumference to explore the effect of adjusting for BMI when the abdominal obesity component was omitted from the outcome score.

## Results

**Fig. 1:**
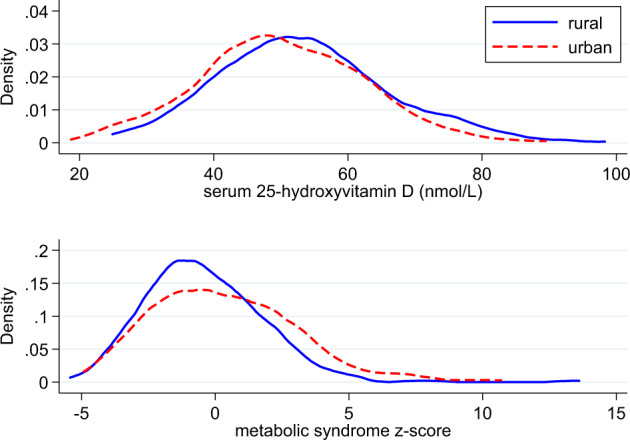
Figure showing urban-rural right shift in the distribution of the metabolic syndrome score and urban-rural left shift in the distribution of serum 25(OH)D concentrations. Distribution of serum 25(OH)D concentrations and metabolic syndrome score by rural and urban residence (Cameroon study, *n* = 574).

The descriptive characteristics of participants stratified by sex and residential site are presented in the supplementary material (Supplementary Tables S1 and S2). Of the 586 participants (273 rural and 313 urban) with mean age (±SD) 38.3 ± 8.6 years, 63.5% were women. Individual cardiometabolic risk factors: waist circumference, BMI, systolic and diastolic blood pressure and HDL cholesterol were higher in urban compared to rural residents. The mean of the metabolic syndrome score was higher in urban residents (0.36 ± 2.7) than rural residents (−0.41 ± 2.27), *p* = 0.0003. This is depicted by the rural-to-urban right shift in the distribution of the metabolic syndrome score (Fig. 1).

Mean serum 25(OH)D was 51.7 ± 12.5 nmol/L with higher concentrations in men (53.5 ± 13.8 nmol/L) than women (50.6 ± 11.6 nmol/L), *p* = 0.008 and rural (53.4 ± 12.8 nmol/L) compared to urban (50.2 ± 12.1 nmol/L) participants (*p* = 0.002). The rural-urban difference was consistent throughout the seasons except during the heavy rainy season when urban participants had higher concentrations of 25(OH)D. There was a rural-to-urban left shift in the distribution of serum 25(OH)D concentrations (Fig. 1). 4.4% of participants had serum 25(OH)D concentrations that were defined as deficient (<30 nmol/L), 41.3% had inadequate but not deficient concentrations (30−49.9 nmol/L), 50.2% had sufficient concentrations (50−74.9 nmol/L) [23], and 4.1% had optimal concentrations (≥75 nmol/L).

Education level, measures of adiposity (waist circumference, BMI and body fat), alcohol intake, FBG, PAEE and sedentary time were all strongly associated with serum 25(OH)D concentrations. There was no evidence of a linear trend in blood pressure, lipids and the metabolic syndrome score across quartiles of serum 25(OH)D (Tables 1 and 2).

**Table 1:** Sociodemographic characteristics by quartiles of serum 25(OH)D concentrations (Cameroon study: *n* = 586).

Characteristics	Quartiles of serum 25(OH)D				*p* for linear trend
	Q1	Q2	Q3	Q4	
25(OH)D (nmol/L)	23.3−44.0	44.1−51.2	51.3−59.8	60.0−98.3	
Sex, *n*(%)					
Female	103 (70.1)	91 (61.9)	95 (65.1)	83 (56.9)	0.04
Age (years)	39.4 ± 8.5	37.4 ± 9.0	38.3 ± 8.3	38.05 ±8.6	0.32
Education, *n*(%)					
(completed)	24 (16.4)	22 (14.9)	29 (19.9)	28 (19.2)	0.003
<primary school	60 (41.1)	58 (39.5)	61 (41.8)	80 (54.8)	
Primary school	39 (26.7)	41 (27.9)	39 (26.7)	28 (19.2)	
Secondary and/or high School University	23 (15.8)	26 (17.7)	17 (11.6)	10 (6.8)	
Smoking status, *n*(%)					
Never	113 (76.9)	114 (77.6)	117 (80.1)	102 (75.0)	0.57
Past	23 (15.7)	18 (12.2)	19 (13.0)	17 (12.5)	
Current smoker	11 (7.4)	15 (10.2)	10 (6.9)	17 (12.5)	
Alcohol intake, *n*(%)					
Never	22 (14.9)	18 (12.2)	15 (10.3)	11 (7.5)	0.001
Past	25 (17.0)	12 (8.2)	5 (3.4)	14 (9.6)	
Current	100 (68.1)	117 (79.6)	126 (86.3)	121 (82.9)	
PAEE (KJ/Kg/day)	43.8 ± 22.4	51.1 ± 22.7	51.1 ± 22.7	55.4 ± 24.7	<0.001
Sedentary time (min/day)	999.8 ± 159.6	947.9 ± 146.32	941.6 ± 150.5	941.6 ± 150.5	0.001
LPA time (min/day)	337.5 ± 112.1	375.8 ± 105.9	375.8 ± 105.9	376.0 ± 98.7	0.007
MVPA time (min/day)	102.7 ± 83.7	114.2 ± 76.7	122.6 ± 85.4	130.4 ± 97.1	0.008
GPAQ PAEE (KJ/Kg/day)	13.3 (3.5−104.9)	23.7 (3.3−112.4)	27.2 (4.2−106.6)	27.9 (3.5−141.1)	0.036
GPAQ work (MET-min/week)	0 (0−8640)	240 (0−9600)	0 (0−10080)	600 (0−12240)	0.55
GPAQ leisure (MET-min/week)	0 (0−0)	0(0−0)	0(0−0)	0(0−0)	
GPAQ travel (MET-min/week)	840 (420−1680)	840 (280−3360)	960 (420−3360)	1120 (420−3360)	0.205
Fruits (times/week)	3 (1−5)	2 (1−5)	2 (1−4)	2 (1−5)	0.729
Vegetables (times/week)	4 (2−7)	4 (2−6)	4 (2−7)	4 (2−8)	0.489

*n* = 586, except for PAEE where *n* = 537.

Results are presented as the arithmetic mean ± SD [or median (25−75th percentile) for non-normally distributed variables] or *n* (%). *p*-values for the trend are from a chi-squared test for trend for categorical variables or from a linear regression model for continuous variables, including quartile of serum 25(OH)D as a continuous exposure.

*PAEE* physical activity energy expenditure, *LPA* light physical activity, *MVP*A moderate to vigorous physical activity.

**Table 2:** Metabolic characteristics by quartiles of serum 25(OH)D concentrations (Cameroon study: *n* = 586).

Characteristics	Quartiles of serum 25(OH)D				*p* for linear trend
	Q1	Q2	Q3	Q4	
Waist circumference (cm)	91.3 ± 13.5	86.7 ± 12.4	90.0 ± 11.8	86.5 ± 10.7	0.013
BMI (kg/m^2^)	27.1 ± 6.0	25.7 ± 5.0	26.8 ± 5.3	24.9 ± 4.4	0.004
Body fat (%)	30.6 ± 11.1	27.5 ± 10.7	29.2 ± 11.4	25.6 ± 10.6	0.001
Systolic BP (mmHg)	121.8 ± 19.1	123.3 ± 21.1	121.8 ± 19.5	123.2 ± 23.0	0.74
Diastolic BP (mmHg)	76.6 ± 12.2	75.8 ± 14.3	77.5 ± 12.3	75.9 ± 14.6	0.94
Fasting BG (mmol/L)	5.0 ± 1.63	4.8 ± 1.49	4.74 ± 1.32	4.55 ± 0.74	0.004
2 h BG (mmol/L)	6.42 ± 1.88	6.37 ± 1.80	6.25 ± 1.95	6.04 ± 1.73	0.065
Fasting insulin (pmol/L)	22.0 (13.3− 32.3)	19.3 (11.7−33.6)	24.1 (10.6−43.1)	19.8 (12.1−30.4)	0.83
HOMA-IR index	0.75 (0.45−1.29)	0.7 (0.37−1.19)	0.78 (0.33−1.62)	0.69 (0.37−1.04)	0.59
Total cholesterol (mmol/L)	3.78 ± 0.89	3.95 ± 0.96	3.86 ± 0.94	3.80 ± 1.1	0.96
HDL cholesterol (mmol/L)	1.23 ± 0.32	1.25 ± 0.32	1.19 ± 0.30	1.21 ± 0.37	0.28
LDL cholesterol (mmol/L)	2.14 ± 0.78	2.34 ± 0.84	2.27 ± 0.78	2.21 ± 0.94	0.60
Triglycerides (mmol/L)	0.73 (0.59−0.98)	0.74 (0.58−0.95)	0.76 (0.58−0.96)	0.74 (0.6−0.91)	0.51
CRP (mg/L)	4.94 (2.42−9.8)	4.6 (2.55−8.59)	4.61 (2.46−8.35)	5.28 (2.65−8.37)	0.64
Adiponectin (µg/mL)	5.41 (3.62−7.81)	5.58 (3.7−7.76)	3.5 (4.67−6.88)	4.15 (6.03−8.36)	0.54
Metabolic syndrome score	0.39 ± 2.94	−0.27 ± 2.42	0.19 ± 2.39	−0.31 ± 2.31	0.087

(*n* = 586, except for metabolic syndrome score, triglycerides and HDL where *n* = 574; 2 h glycaemia (*n* = 577) HOMA-IR (*n* = 582)).

Results are presented as the arithmetic mean ± SD [or median (25−75th percentile) for non-normally distributed variables] or *n* (%). *p*-values for the trend are from a chi-squared test for trend for categorical variables or from a linear regression model for continuous variables, including quartile of serum 25(OH)D as a continuous exposure.

*BMI* body mass index, *BP* blood pressure, *BG* blood glucose, *CRP* C-reactive protein.

We also examined sociodemographic, behavioural and anthropometric correlates of serum 25(OH)D in models adjusted for age and sex and stratified by rural/urban site (Supplementary Table S3). Variables that had a positive association with serum 25(OH)D were male sex, PAEE (with stronger associations for objectively measured PAEE) and season of blood draw (light rain in rural and heavy rain in urban). Time spent being sedentary, measures of adiposity (BMI, body fat and waist circumference), and level of education were inversely associated with serum 25(OH)D concentrations.

The associations between serum 25(OH)D and outcomes are shown in Table 3. There was an inverse association between serum 25(OH)D and the metabolic syndrome score in the unadjusted model (β-coefficient −0.30, 95% CI −0.55 to −0.05 per 1 SD of 25(OH)D). This was attenuated and became non-significant after adjusting for age, smoking status, alcohol intake and education level (model 2). For the glycemic markers, an inverse association between 25(OH)D and FBG was observed, which remained significant after adjusting for age, sex, smoking status, alcohol intake and education level, residential site, season of data collection, BMI and PAEE (β-coefficient -0.17, 95% CI −0.29 to −0.06 per 1 SD of 25(OH)D) (model 5). Serum 25(OH)D was also inversely associated with 2-h glucose and HOMA-IR but this was attenuated and became non-significant after adjusting for confounders (model 2 for HOMA-IR and model 4 for 2-h glucose).

**Table 3:** Associations between serum 25(OH)D concentrations and cardiometabolic risk factors (Cameroon study).

Difference in outcome per 12.5 nmol/L (1 SD) of 25(OH)D	Model 1		Model 2		Model 3		Model 4		Model 5	
	β (95% CI)	*p*-value	β (95% CI)	*p*-value	β (95% CI)	*p*-value	β (95% CI)	*p*-value	β (95% CI)	*p*-value
Metabolic syndrome score (*n* = 528)	−0.30 (−0.55 to −0.05)	0.020	−0.23 (−0.47 to 0.01)	0.065	−0.18 (−0.42 to 0.05)	0.130	0.06 (−0.27 to 0.15)	0.587	0.03 (−0.24 to 0.18)	0.783
Fasting BG (mmol/L, *n* = 537)	−0.21 (−0.34 to −0.08)	0.0003	−0.18 (−0.29 to −0.07)	0.002	−0.19 (−0.30 to −0.08)	0.001	−0.17 (−0.29 to −0.06)	0.003	−0.17 (−0.29 to −0.06)	0.003
2-h BG (mmol/L, *n* = 530)	−0.20 (−0.34 to −0.05)	0.009	-0.17 (-0.32 to -0.02)	0.027	−0.16 (−0.30 to −0.01)	0.041	−0.14 (−0.29 to 0.01)	0.063	−0.14 (−0.29 to 0.01)	0.061
HOMA-IR index (*n* = 534)	−0.12 (−0.19 to −0.04)	0.003	−0.07 (−0.14 to 0.02)	0.128	−0.06 (−0.14 to 0.02)	0.127	−0.03 (−0.11 to 0.05)	0.425	−0.02 (−0.10 to 0.06)	0.569

*BG* blood glucose, *HOMA-IR* homeostatic model assessment of insulin resistance.

Model 1: Unadjusted.

Model 2: Adjusted for age, sex, smoking status, alcohol intake and education level.

Model 3: Model 2 + residential site (4 sites) and season (4 seasons).

Model 4: model 3 + BMI (continuous).

Model 5: model 4 + PAEE (continuous).

There was evidence of interaction between serum 25(OH)D and urban/rural residential site on the metabolic syndrome score (*p*-value for interaction = 0.016 in model 5). In model 3 adjusting for age, sex, smoking status, alcohol intake, education level, residential site and season, a significant inverse association between 25(OH)D and metabolic syndrome score was observed in the urban subgroup (β-coefficient −0.52, 95% CI −0.87 to −0.16) but not in the rural subgroup (+0.21, −0.06 to +0.49 per 1 SD of 25(OH)D) (Supplementary Table S4). However, the inverse association in the urban subgroup was attenuated and became non-significant after adjusting for BMI and objectively-measured physical activity. No evidence of interaction was observed by sex or obesity status (*p* > 0.05).

Results were unchanged in the sensitivity analyses in which we a) used multiple imputation to investigate the impact of missing data (Supplementary Table S5); b) replaced BMI by body fat in model 4 and 5; and c) adjusted for self-reported fruit and vegetable intake as a proxy for overall dietary quality in model 5. There was no association between the serum 25(OH)D and the metabolic syndrome computed without the abdominal obesity component.

## Discussion

The present analysis based on a cross-sectional population-based study of adults in rural and urban Cameroon demonstrates inverse associations between vitamin D status, assessed by 25(OH)D concentrations, and a composite score of the metabolic syndrome and glycemic markers. The association with fasting glucose was independent of age, sex, education, smoking, alcohol intake, season of data collection, BMI and objectively measured physical activity. Although adequate vitamin D status is crucial for bone and muscular health, the association with cardiometabolic health is still debated. Our findings provide evidence that vitamin D may modulate glucose homoeostasis in people in sub-Saharan Africa who tend to have low concentrations of 25(OH)D.

In this study, 45.7% of the participants had vitamin D insufficiency (25(OH)D < 50 nmol/L), which is higher than the prevalence reported in Europe (40.4%) [12] and the US (23.3%) [25]. Differences in assay method for 25(OH)D limit our ability to compare the prevalence of vitamin D insufficiency with previous studies in Africa. A meta-analysis of vitamin D status in Africa reported a prevalence of vitamin D insufficiency (<50 nmol/L) of 34% with a high variation between countries ranging from 4% in Ghana to 99% in Sudan [10]. However, the reported prevalence is likely to be underestimated as over 90% of the studies used immunoassay-based techniques. Immunoassays have been shown to yield 25(OH)D concentrations that are ~50% higher than LC-MS/MS due to the high cross-reactivity with 24,25(OH)D_2_ [26]. While sunshine is abundant in Africa, several factors may account for the high prevalence of low vitamin D status in our population, such as dark skin pigmentation, limited skin exposure to sunshine due to clothing and cultural practices, low dietary calcium intakes and a heavy burden of infectious disease [27].

In this study, we observed an inverse association between vitamin D status and metabolic syndrome score which became non-significant after adjusting for sociodemographic and health-behavioural characteristics, which is consistent with previous observational studies [4, 13, 14, 28]. Other studies found an inverse association between vitamin D status and metabolic syndrome independently of potential confounders [2, 29, 30]. However, these associations could be the result of residual confounding due to the imprecise measurement of covariates such as physical activity and adiposity. Most previous studies either did not adjust for physical activity or adjusted only self-reported physical activity, thus creating the potential for residual confounding even after adjustment. We observed an inverse association with fasting glucose, which remained unchanged after adjusting for potential confounders, including adiposity and physical activity. This finding supports previous research suggesting that the association between vitamin D and metabolic syndrome risk is largely driven by the glycemic parameters [4]. A meta-analysis of 22 studies showed that lower 25(OH)D concentration was associated with higher diabetes risk [31].

Physical activity has been shown to be a strong correlate of vitamin D status, with similar associations for self-reported outdoor and indoor physical activity [32]. This suggests the association between vitamin D and physical activity may be independent of sunlight exposure [33]. Our results show that the association between serum 25(OH)D and objectively measured PAEE was stronger than that with self-reported physical activity. Higher BMI, waist circumference and body fat were all associated with lower 25(OH)D concentrations in our analyses. Vitamin D is a fat-soluble vitamin and may get sequestrated in adipose tissues leading to lower vitamin D bioavailability [34]. This may also explain the lower 25(OH)D concentrations in women. We accounted for adiposity by using BMI and our results were unchanged in a sensitivity analysis in which we replaced BMI with body fat % measured by bioimpedance analysis. After adjusting for adiposity measures, the inverse association of serum 25(OH)D and metabolic syndrome score was further attenuated to the null, in particular in the urban population, suggesting adiposity is an important source of confounding in the inverse association observed in the unadjusted analysis.

Mechanisms by which vitamin D may reduce the risk of the development of cardiometabolic diseases have not been fully elucidated. Vitamin D appears to play a role directly or indirectly in insulin secretion, insulin sensitivity and systemic inflammation. Evidence from animal studies suggests a direct effect of 1,25(OH)_2_D, the active form of vitamin D, on insulin secretion via the activation of vitamin D receptors expressed in the pancreatic β-cells and on insulin sensitivity by stimulating the expression of insulin receptors in peripheral target tissues [35]. Indirectly, low vitamin D status could stimulate parathyroid hormone release, which has been shown to be associated with insulin resistance. Furthermore, low vitamin D status appears to activate macrophages, suggesting a role in inflammatory processes. The identification of vitamin D receptors in over 30 different tissues has led to the hypothesis of its effect on several health outcomes [36]. However, in a mendelian randomization study, genetically predicted higher 25(OH)D concentration was not associated with type 2 diabetes risk [37]. Our findings of an inverse association between vitamin D status and fasting glycemia and the null association with HOMA-IR suggest an effect via insulin secretion rather than insulin resistance.

Most RCTs have consistently shown a null association between vitamin D supplementation and cardiometabolic endpoints, including a recent trial examining intermediate endpoints (high-sensitivity CRP and N-terminal pro-B-type natriuretic peptide) [8, 9, 38–41]. However, some of these trials were of small sample size and short duration [40], used a low dose of vitamin D [41], were only powered to detect large effect sizes [8], and were not designed to study cardiometabolic diseases as primary endpoints [41]. In addition, participants in most of these trials already had an adequate vitamin D status at baseline [8, 9]. In the vitamin D and type 2 diabetes study, an RCT specifically designed to test diabetes as an endpoint, vitamin D supplementation lowered diabetes risk in people with low baseline vitamin D status only (< 30 nmol/L).

Considering the evidence from reviews suggesting that vitamin D status is worse in some parts of Africa compared with other parts of the world, understanding the link between vitamin D status and metabolic risk factors in this part of the world is important [15]. If supported by further evidence from prospective studies and RCTs, strategies to improve vitamin D status in populations with a high burden of vitamin D deficiency may provide a cheap and feasible public health approach to improve cardiometabolic health in these populations.

### Strengths and limitations

To our knowledge, this is the first population-based study from sub-Saharan Africa to examine the association between vitamin D status and cardiometabolic risk factors using a metabolic syndrome score calculated as a continuous variable. The metabolic syndrome score has emerged as a valid and alternative tool to the dichotomous metabolic syndrome variable [21] and the use of a continuously distributed score maximises statistical power. We used the gold standard method to measure serum 25(OH)D leading to greater precision in the exposure. In addition, we adjusted for a wide range of potential confounders, including objective measures of physical activity and adiposity. Nonetheless, some limitations warrant attention. The sample size was small, which limited our ability to conduct subgroup analysis and our findings may not be generalizable to a population outside this geographical region. Moreover, the cross-sectional design of our study limits causal inference. It is possible that poor metabolic health could have led to dietary changes, reduced sun exposure, physical activity, or increased inflammation, all of which could affect serum 25(OH)D concentrations (reverse causality). Data were not available on sun exposure or dietary intake of vitamin D from natural, fortified food or dietary supplements. However, it is widely known that the contribution of vitamin D from natural food sources is low. Because there is no mandatory vitamin D fortification of foods and the use of dietary supplements among healthy African adults is low, sunlight exposure is likely to be the main source of vitamin D in this population [42]. The proportion of participants in our study with serum 25(OH)D concentrations below 30 nmol/L or above 75 nmol/L was low, which limits our ability to investigate the relationship at the extremes of the range. Future prospective studies and trials are needed to confirm the role of vitamin D on metabolic health in populations at high risk of vitamin D insufficiency.

## Conclusion

In conclusion, this population-based study showed an inverse association between serum 25-hydroxyvitamin D and a composite score of the metabolic syndrome, which was confounded by socio-demographic characteristics. The inverse association of 25(OH)D with fasting glucose was independent of age, sex, education, smoking, alcohol intake, season of data collection, BMI and objectively measured physical activity. This suggests that public health interventions to improve vitamin D status may improve glucose metabolism in this population and warrant further investigation in prospective studies.

## Supplementary information

Table S1, Table S2, Table S3, Table S4, Table S5

## Data Availability

The data that support the findings of this study are available from the corresponding author upon reasonable request.
