# Supplementary material for: Association between circulating 25-hydroxyvitamin D and cardiometabolic risk factors in adults in rural and urban settings
Source: Nutr Diabetes. 2022 Jul 29;12:34. doi: 10.1038/s41387-022-00215-1 (PMC9338254; doi:10.1038/s41387-022-00215-1)
Supplement: Supplementary file 1 — Table S1, Table S2, Table S3, Table S4, Table S5 [file 41387_2022_215_MOESM1_ESM.docx]

Supplementary material:

Table S1: Socio-demographic characteristics of the study population stratified by sex and urban/rural residence (Cameroon study: n=586)

|  | Women (n=372) | | | Men (n=214) | | |
| --- | --- | --- | --- | --- | --- | --- |
|  | Rural (n=173) | Urban (n=199) | p-value | Rural (n=100) | Urban (n=114) | p-value |
| Age (years) | 39.8±8.1 | 38.6±8.8 | 0.168 | 37.3±8.3 | 36.3±8.9 | 0.368 |
| Education (years) | 8.0±4.1 | 11.8±5.2 | <0.001 | 9.0±4.5 | 13.2±5.2 | <0.001 |
| Education level, n (%)  < Primary education  Primary education  Secondary and high school  University | 53(30.6)  93(53.8)  24(13.9)  3(1.7) | 24(12.0)  75(37.7)  66(33.2)  34(17.1) | < 0.001 | 22(22.2)  57(57.6)  16(16.2)  4(4.0) | 4(3.5)  34(29.8)  41(36.0)  35(30.7) | < 0.001 |
| Alcohol intake, n (%)  Never  Past  Current | 26(15.0)  17(9.8)  173(75.2) | 24(12.1)  26(13.1)  149(74.8) | 0.485 | 10(10.0)  03(3.0)  87(87.0) | 06(5.3)  10(8.7)  98(86.0) | 0.104 |
| Smoking status, n (%):  Never  Past smoker  Current smoker | 161(93.1)  11(6.4)  1(0.6) | 182(91.5)  16(8.0)  1(0.5) | 0.820 | 57(57.0)  21(21.0)  22(22.0) | 54(47.4)  31(27.2)  29(25.4) | 0.357 |
| Fruit (times/week) | 3(2-6) | 2(1-5) | 0.108 | 2(1-6) | 2(1-3) | 0.028 |
| Vegetable (times/week) | 6(3-9) | 4(2-6) | <0.0001 | 4(2-6) | 3(2-6) | 0.153 |
| Fruit and vegetable, n (%)  < 3 times/ week  3-6 times/ week  ≥ 7 times per week | 7(4.3)  41(25.2)  115(70.6) | 15(8.2)  74(40.2)  95(51.6) | 0.001 | 12(12.6)  29(30.5)  54(56.8) | 16(14.8)  53(49.1)  39(36.1) | 0.01 |
| PAEE (KJ/Kg/day) | 54.3±20.9 | 38.3±16.6 | <0.0001 | 65.9±26.2 | 52.1±22.6 | 0.0001 |
| Sedentary time (min/day) | 925.1±143.7 | 1016.5±137.3 | <0.0001 | 878.1±154.1 | 961.0±150.9 | 0.0003 |
| LPA time (min/day) | 379.0±95.8 | 333.1±104.1 | 0.0001 | 415.7±97.9 | 366.5±112.4 | 0.002 |
| MVPA time (min/day) | 116.2(69.6-201.6) | 75.3(47.5-113.2) | <0.0001 | 133.1(62.5-202.1) | 94.4(54.6-149.9) | 0.025 |
| GPAQ PAEE (KJ/Kg/day) | 82.5(6.8-178.3) | 8.1(3.4-53.8) | <0.0001 | 35.9(3.2-140.1) | 18.4(3.5-69.8) | 0.22 |
| GPAQ work (MET-min/week) | 7280(0-16320) | 0(0-4800) | <0.0001 | 2120(0-12600) | 0(0-5760) | 0.032 |
| GPAQ leisure (MET-min/week) | 0(0-0) | 0(0-0) |  | 0(0-0) | 0(0-0) |  |
| GPAQ travel (MET-min/week) | 1680(560-3360) | 720(300-1680) | <0.0001 | 840(390-4920 | 840(280-2520) | 0.328 |

Results are presented as arithmetic mean [or median (25th-75th percentile) for non-normally distributed variables] or n (%). p-values are from a t-test for normally distributed continuous variables (or Mann Whitney test for non-normally distributed variables) and from a chi squared test for categorical variables.

PAEE, physical activity energy expenditure; LPA, light physical activity; MVPA, moderate to vigorous physical activity

Table S2: Metabolic characteristics of the study population stratified by sex and urban/rural residence (Cameroon study: n=586)

|  | Women (n=372) | | | Men (n=214) | | |
| --- | --- | --- | --- | --- | --- | --- |
|  | Rural (n=173) | Urban (n=199) | p-value | Rural (n=100) | Urban (n=114) | p-value |
| BMI (kg/m2) | 24.9±4.9 | 29.2±5.5 | <0.0001 | 23.09±3.23 | 25.3±4.0 | <0.0001 |
| BMI (kg/m2)  Continuous  <25  25-29.9  ≥ 30 | 104(60.1)  41(23.7)  28(16.2) | 50(25.1)  56(28.1)  93(46.8) | <0.001 | 75(75.0)  22(22.0)  03(3.0) | 67(58.8)  36(31.6)  11(9.7) | 0.023 |
| Waist circumference (cm) | 85.2±11.5 | 94.3±12.9 | <0.0001 | 82.9±7.9 | 89.2±11.3 | <0.0001 |
| Central obesity (%) | 114 (65.9) | 168(84.4) | <0.0001 | 9(9.0) | 31(27.2) | 0.001 |
| Waist to hip ratio | 0.84±0.08 | 0.85±0.07 | 0.88 | 0.86±0.05 | 0.87±0.06 | 0.11 |
| Body fat (%) | 30.2±8.3 | 37.4±7.6 | <0.0001 | 15.8±5.5 | 20.1±7.0 | <0.0001 |
| Systolic blood pressure (mmHg) | 117.1±18.8 | 123.8±21.9 | 0.002 | 120.5±15.1 | 130.4±22.9 | 0.0003 |
| Diastolic Blood Pressure (mmHg) | 74.2±12.0 | 79.6±13.7 | <0.0001 | 72.3±11.9 | 78.0±14.6 | 0.002 |
| Fasting blood glucose (mmol/L) | 4.81±1.39 | 4.86±1.08 | 0.682 | 4.72±1.49 | 4.63±1.55 | 0.676 |
| 2-hour blood glucose (mmol/L) | 6.23±1.74 | 6.61±1.93 | 0.053 | 5.94±1.55 | 6.02±1.88 | 0.737 |
| Total cholesterol (mmol/L) | 3.84±0.95 | 4.02±0.98 | 0.08 | 3.60±0.90 | 3.80±0.90 | 0.318 |
| LDL cholesterol (mmol/L) | 2.26±0.83 | 2.36±0.85 | 0.23 | 2.07±0.79 | 2.15±0.85 | 0.363 |
| HDL cholesterol (mmol/L) | 1.18±0.33 | 1.27±0.32 | 0.017 | 1.19±0.35 | 1.23±0.31 | 0.362 |
| Triglycerides (mmol/L) | 0.78(0.63-1.01) | 0.71(0.57-0.90) | 0.026 | 0.70(0.58-0.94) | 0.73(0.56-0.99) | 0.876 |
| Fasting insulin (pmol/L) | 22.3(14.2-39.1) | 24.7(13.8-37.7) | 0.747 | 14.8(6.5-29.3) | 17.2(8.1-28.9) | 0.514 |
| CRP (mg/L) | 5.26(2.61-8.41) | 4.58(2.63-8.31) | 0.61 | 5.69(2.75-10.16) | 4.17(2.05-8.24) | 0.048 |
| Adiponectin (µg/ml) | 6.82(4.67-9.34) | 5.87(3.72-7.96) | 0.0005 | 4.34(3.04-6.41) | 4.42(3.07-5.93) | 0.986 |
| HOMA-IR index | 0.77(0.47-1.46) | 0.87(0.49-1.32) | 0.639 | 0.49(0.20-1.01) | 0.59(0.23-1.05) | 0.502 |
| 25(OH)D (nmol/L) | 51.9±11.8 | 49.5±11.4 | 0.044 | 55.9±14.0 | 51.4±13.3 | 0.018 |
| Metabolic syndrome score | -0.32±2.25 | 0.35±2.67 | 0.01 | -0.56±2.30 | 0.37±2.82 | 0.01 |

Results are presented as arithmetic mean [or median (25th-75th percentile) for non-normally distributed variables] or n (%). p-values are from a t-test for normally distributed continuous variables (or Mann Whitney test for non-normally distributed variables) and from a chi squared test for categorical variables.

BMI, body mass index; CRP, C-reactive protein. Central obesity was defined as waist circumference ≥ 94 cm in men and 80 cm in women. Obesity using BMI was defined as BMI ≥ 30 Kg/m^2^

Table S3: Socio demographic and anthropometric correlates of vitamin D status (Cameroon study: n=586)

| Correlates | Total (586) | | Rural (n=273) | | Urban (n=313) | |
| --- | --- | --- | --- | --- | --- | --- |
|  | β (95% CI) | p-value | β (95% CI) | p-value | β (95% CI) | p-value |
| Age (years) | -0.06(-0.18 to 0.06) | 0.32 | -0.04(-0.22 to 0.15) | 0.68 | -0.1(-0.25 to 0.05) | 0.21 |
| Male (vs female) | 2.72(0.60 to 4.84) | 0.012 | 3.84(0.67 to 7.0) | 0.018 | 1.69(-1.13 to 4.51) | 0.24 |
| Education level  < Primary education (ref)  Primary education  Secondary and high school  University | -0.79(-3.65 to 2.05)  -4.29(-7.45 to -1.14)  -7.29(-11.06 to -3.52) | 0.58  0.008  <0.001 | 0.84(-2.78 to 4.46)  -3.60(-8.57 to 1.37)  -3.59(-13.40 to 6.62) | 0.649  0.155  0.472 | -3.01(-8.05 to 2.02)  -4.99(-10.1 to 0.10)  -8.37(-13.88 to -2.86) | 0.240  0.06  0.003 |
| Smoking status  Never smoked (ref)  Former  Current | -1.85(-4.9 to 1.30)  -1.09(-5.05 to 2.87) | 0.25  0.59 | 0.58(-4.36 to 5.52)  -2.46(-8.51 to 3.59) | 0.818  0.424 | -2.95(-7.0 to 1.11)  0.45(-4.76 to 5.67) | 0.154  0.86 |
| Alcohol intake  Never (ref)  Former  Current | -1.49(-5.91 to 2.93)  3.20(0.14 to 6.42) | 0.508  0.051 | 3.53(-3.40 to 10.46)  5.12(0.63 to 9.59) | 0.317  0.025 | -4.23(-10.08 to 1.63)  1.58(-3.04 to 6.19) | 0.156  0.502 |
| Season of blood draw  Short dry (ref)  Long dry  Light rain  Heavy rain | 1.15(-1.17 to 3.47)  -2.97(-0.33 to 6.26)  4.32(0.99 to 7.66) | 0.332  0.077  0.011 | -0.17(-3.7 to 3.37)  6.75(1.45 to 12.04)  1.24(-3.28 to 5.78) | 0.926  0.013  0.589 | 2.26(-0.87 to 5.38)  0.69(-3.44 to 4.82)  7.55(2.56 to 12.54) | 0.156  0.741  0.003 |
| Fruits (times/week) | 0.06(-0.23 to 0.35) | 0.71 | -0.07(-0.46 to 0.31) | 0.706 | 0.09(-0.361 to 0.55) | 0.697 |
| Vegetables (times/week) | 0.30(-0.05 to 0.56) | 0.02 | 0.37(-0.01 to 0.72) | 0.043 | 0.08(-0.30 to 0.47) | 0.669 |
| PAEE (KJ/kg/day) | 0.1(0.05 to 0.14) | <0.001 | 0.02(-0.05 to 0.08) | 0.656 | 0.17(0.09 to 0.22) | <0.001 |
| Objective sedentary time (min/day) | -0.01( -0.02 to -0.005) | 0.001 | -0.001(-0.01 to 0.01) | 0.859 | -0.02 (-0.03 to -0.01) | 0.001 |
| Objective LPA (min/day) | 0.01(-0.001 to 0.02) | 0.027 | 0.002(-0.02 to 0.02) | 0.834 | 0.01(-0.001 to 0.03) | 0.061 |
| Objective MVPA (min/day) | 0.02(0.01 to 0.03) | 0.004 | 0.001(-0.02 to 0.02) | 0.949 | 0.03(0.01 to 0.05) | 0.001 |
| GPAQ PAEE (KJ/Kg/day) | 0.02(0.007 to 0.03) | 0.002 | 0.01(-0.005 to 0.03) | 0.157 | 0.02(0.001 to 0.04) | 0.043 |
| GPAQ work (MET-h/week) | 0.01(0.003 to 0.02) | 0.007 | 0.005(-0.005 to 0.015) | 0.348 | 0.01(0.003 to 0.025) | 0.044 |
| GPAQ leisure (MET-h/week) | 0.04(-0.003 to 0.07) | 0.07 | 0.04(-0.006 to 0.09) | 0.087 | 0.02(-0.04 to 0.09) | 0.410 |
| GPAQ travel (MET-h/week) | 0.03(0.005 to 0.06) | 0.019 | 0.03(-0.006 to 0.07) | 0.119 | 0.02(-0.03 to 0.06) | 0.427 |
| BMI (kg/m^2^)  Continuous  <25 (ref)  25-29.9  ≥ 30 | -0.31(-0.51 to -0.11)  -2.23(-4.68 to -0.23)  -3.59(-6.30 to -0.89) | 0.003  0.076  0.009 | 0.008(-0.34 to 0.36)  -1.36(-5.05 to 2.34)  -0.58(-5.58 to 4.42) | 0.964  0.470  0.820 | -0.40(-0.67 to -0.12)  -2.23(-5.71 to 1.25)  -3.91(-7.59 to -0.23) | 0.005  0.208  0.037 |
| Body fat (%) | -0.21(-0.34 to -0.08) | 0.002 | 0.004(-0.21 to 0.21) | 0.970 | -0.31(-0.50 to -0.11) | 0.002 |
| Waist circumference (cm) | -0.13(-0.21 to -0.04) | 0.005 | 0.05(-0.10 to 0.20) | 0.498 | -0.18(-0.30 to -0.07) | 0.002 |

β-coefficients represent the difference in serum 25(OH)D in nmol/L per a unit difference in the predictor. Estimates are adjusted for age and sex (except for age adjusted for sex only and sex adjusted for age only)

PAEE, physical activity energy expenditure; LPA, Light physical activity; MVPA, moderate to vigorous PA; BMI, body mass index; BG, blood glucose

Table S4: Associations between serum 25(OH)D concentrations and metabolic syndrome score stratified by rural/urban residential site (Cameroon study, n=528)

| Difference in metabolic syndrome score per 1 SD of serum 25(OH)D concentrations | Rural (n=245) | | Urban (283) | |
| --- | --- | --- | --- | --- |
|  | β (95% CI) | p-value | β (95% CI) | p-value |
| Model 1 | 0.22(-0.04 to -0.48) | 0.097 | -0.68(-1.06 to -0.29) | 0.001 |
| Model 2 | 0.22(-0.04 to -0.48) | 0.101 | -0.51(-0.87 to -0.15) | 0.005 |
| Model 3 | 0.21(-0.06 to -0.49) | 0.130 | -0.52(-0.87 to -0.16) | 0.005 |
| Model 4 | 0.23(-0.02 to 0.47) | 0.074 | -0.27(-0.60 to 0.05) | 0.99 |
| Model 5 | 0.23(-0.01 to -0.47) | 0.06 | -0.23(-0.55 to 0.09) | 0.163 |

Model 1: Unadjusted

Model 2: Adjusted for age, sex, smoking, alcohol intake, education level

Model 3: Model 2 + residential site (2 sites), season (4 seasons)

Model 4: model 3 + BMI (continuous)

Model 5: model 4 + PAEE (continuous)

Table S5: Associations between serum 25(OH)D concentrations and cardiometabolic risk factors using multiple imputation for missing data (Cameroon study: n=586)

| Difference in outcome per 12.47 nmol/L (1 SD) change in 25(OH)D concentration | Model 1 | | Model 2 | | Model 3 | | Model 4 | | Model 5 | |
| --- | --- | --- | --- | --- | --- | --- | --- | --- | --- | --- |
|  | β (95% CI) | p-value | β (95% CI) | p-value | β (95% CI) | p-value | β (95% CI) | p-value | β (95% CI) | p-value |
| Metabolic syndrome score | -0.24 (-0.47 to 0.01) | 0.039 | -0.19 (-0.41 to 0.03) | 0.098 | -0.16 (-0.38 to 0.06) | 0.151 | -0.04 (-0.24 to 0.15) | 0.664 | -0.01 (-0.21 to 0.18) | 0.884 |
| Fasting BG (mmol/L, | -0.18 (-0.30 to -0.06) | 0.003 | -0.16 (-0.26 to -0.05) | 0.003 | -0.17 (-0.27 to -0.06) | 0.002 | -0.15 (-0.25 to -0.04) | 0.008 | -0.15 (-0.25 to -0.04) | 0.007 |
| 2-h BG (mmol/L) | -0.19 (-0.35 to -0.04) | 0.013 | -0.14 (-0.29 to 0.003) | 0.055 | -0.15 (-0.30 to -0.003) | 0.054 | -0.13 (-0.28 to 0.02) | 0.096 | -0.13 (-0.28 to 0.02) | 0.082 |
| HOMA-IR index | -0.11 (-0.17 to -0.04) | 0.004 | -0.06 (-0.13 to 0.02) | 0.139 | -0.05 (-0.12 to 0.03) | 0.203 | -0.02 (-0.10 to 0.06) | 0.555 | -0.01 (-0.09 to 0.07) | 0.778 |

Model 1: Unadjusted

Model 2: Adjusted for age, sex, smoking, alcohol intake, education level

Model 3: Model 2 + residential site (4 sites) and season (4 seasons)

Model 4: model 3 + BMI (continuous)

Model 5: model 4 + PAEE (continuous)
